# Increased Risk of Cancer in relation to Gout: A Review of Three Prospective Cohort Studies with 50,358 Subjects

**DOI:** 10.1155/2015/680853

**Published:** 2015-10-04

**Authors:** Weijie Wang, Donghua Xu, Bin Wang, Shushan Yan, Xiaochen Wang, Yin Yin, Xuehao Wang, Beicheng Sun, Xiaoyang Sun

**Affiliations:** ^1^Department of Neurosurgery, Huai'an First People's Hospital Affiliated to Nanjing Medical University, 6 Beijing Road West, Huai'an 223300, China; ^2^Department of Rheumatology, The First Affiliated Hospital, Nanjing Medical University, Nanjing 210029, China; ^3^Liver Transplantation Center of the First Affiliated Hospital and State Key Laboratory of Reproductive Medicine, Nanjing Medical University, Nanjing 210002, China; ^4^Department of Endocrinology, The Affiliated Hospital of Medical College, Qingdao University, Qingdao 266003, China; ^5^Department of Surgical Oncology, The Eighty-First Hospital of People's Liberation Army, Nanjing 210002, China

## Abstract

Gout is a common inflammatory disease characterized by acute arthritis and hyperuricemia. A number of epidemiological studies have suggested the critical role of gout in carcinogenesis. The aim of this study was to estimate the association between gout and cancer risk by meta-analysis of all relevant studies published to date. A comprehensive literature search in PubMed and Embase databases from their inception up to July 1, 2014, was performed to identify eligible studies. The strength for relationship between gout and the risk of different cancers was evaluated by calculating pooled relative risks (RRs) with 95% confidence intervals (95% CIs). All analyses were carried out by STATA 12.0 software. Gout patients were at an increased risk of cancer, particularly urological cancers, digestive system cancers, and lung cancer. No such significant association between gout and the risk of breast or brain cancers was observed. Sensitivity analysis did not materially alter the pooled results. Gout is a risk factor of cancer, particularly that of urological cancers, digestive system cancers, and lung cancer. The pooled data further support the hypothesis of a link between gout and carcinogenesis.

## 1. Introduction

Gout is a common inflammatory disease characterized by acute arthritis and hyperuricemia due to disorder of purine metabolism [1]. It is caused by monosodium urate crystal deposition in tissues leading to arthritis, soft tissue masses, kidney stones, and urate nephropathy. Attacks of pain, erythema, and swelling of one or a few joints in the lower extremities are prominent clinical manifestations of acute gout. Higher intakes of red meat, fructose and beer, alcohol consumption, and genetic polymorphisms have been implicated in the development of gout and hyperuricemia [2–4]. The prevalence of gout is increasing worldwide. It is more common in men and strongly age-related. To the best of our knowledge, gout is highly related to obesity, hypertension, diabetes mellitus, chronic kidney, and cardiovascular diseases [1, 2, 5].

During the past few years, the association between cancer and gout as well as hyperuricemia has drawn a lot of attention. High cell turnover can lead to hyperuricemia and tumorigenesis, implicating an underlying link between purine metabolism disorders and cancer [6]. It has been well documented that high serum uric acid levels are independently related to an increased risk of cancer [7–9]. In addition, massive lysis of malignant cells in certain patients with tumor lysis syndrome can result in hyperuricemia, which can predispose patients to renal failure, coronary heart disease, and gout [10, 11]. Interestingly, uric acid has also been hypothesized to play a protective role in carcinogenesis due to its systemic antioxidant properties [12]. Nonetheless, this issue remains controversial. Taken together, all available data support the hypothesis of a link between metabolic syndrome and tumorigenesis.

Although recent epidemiological studies have sparked interest in the hypothesis, the precise association between gout and cancer risk remains obscure. Our study was firstly aimed to investigate this association by meta-analysis of all currently available data.

## 2. Materials and Methods

### 2.1. Search Strategy

We performed a comprehensive literature search in PubMed and Embase databases from their inception up to July 1, 2014, for studies on the association between gout and cancer risk. The following search terms were used: gout, or hyperuricemia; cancer, tumor, or carcinoma; risk, or incidence. The references of retrieved studies were also screened for additional publications. No language restriction was imposed.

### 2.2. Inclusion and Exclusion Criteria

The included studies must meet the inclusion criteria as follows: (1) studies concerning the association between gout and cancer risk; (2) studies in a cohort design; (3) studies with enough information for odds ratio (ORs), relative risks (RRs), or hazard ratios (HRs) with corresponding 95% confidence intervals (95% CIs).

Studies were excluded if they were (1) publications not related to the role of gout in cancer risk; (2) case-only study; (3) case report; (4) reviews; (5) animal studies; (6) studies with overlapping data.

### 2.3. Data Extraction

Two investigators independently extracted available data from all studies as follows: first author's name, year of publication, origins, diagnosis of patients, study design, type of cancers, total sample size, study period, adjusted factors, baseline time, follow-up duration, mean age of gout patients, and RRs or HRs or ORs with corresponding 95% CIs. Disagreements were resolved by consensus.

### 2.4. Statistical Analysis

The strength for relationship between gout and cancer risk was estimated by calculating the pooled RRs with 95% CIs. The between-study heterogeneity was evaluated by Cochran's Q-statistic test and *I* ^2^ test [14, 15]. *P* < 0.05 and *I* ^2^ > 50% implicated potential between-study heterogeneity among studies. The fixed-effects model by Mantel and Haenszel method was used if the between-study heterogeneity was not statistically significant [16]; otherwise, the random-effects model by way of DerSimonian and Laird was applied with regard to significant between-study heterogeneity [17]. Subgroup analyses by different type of cancer and sensitivity analysis were also carried out to assess the association. Begg's funnel plots and Egger's test were conducted to estimate possible publication bias in our study [18, 19]. The STATA 12.0 software (StataCorp, College Station, TX, USA) was used. All *P* values were two-tailed, and *P* < 0.05 suggested statistical significance.

## 3. Results

### 3.1. Characteristics of All Eligible Studies

After a comprehensive literature search, three prospective cohort studies with a total of 50, 358 subjects were retrieved for meta-analysis [6, 13, 20]. The study by Boffetta et al. was divided into two independent studies according to gender. The characteristics of included studies were summarized in Table 1 mainly including the name of the first author, publication year, baseline time, follow-up duration, type of cancer, adjusted factors, and mean age of gout patients. The effect of gout on cancer was primarily evaluated in the development of urological cancers, digestive system cancers, lung cancer, breast cancer, and brain tumors (Table 1).

### 3.2. Association between Gout and Cancer Risk

**Figure 1:**
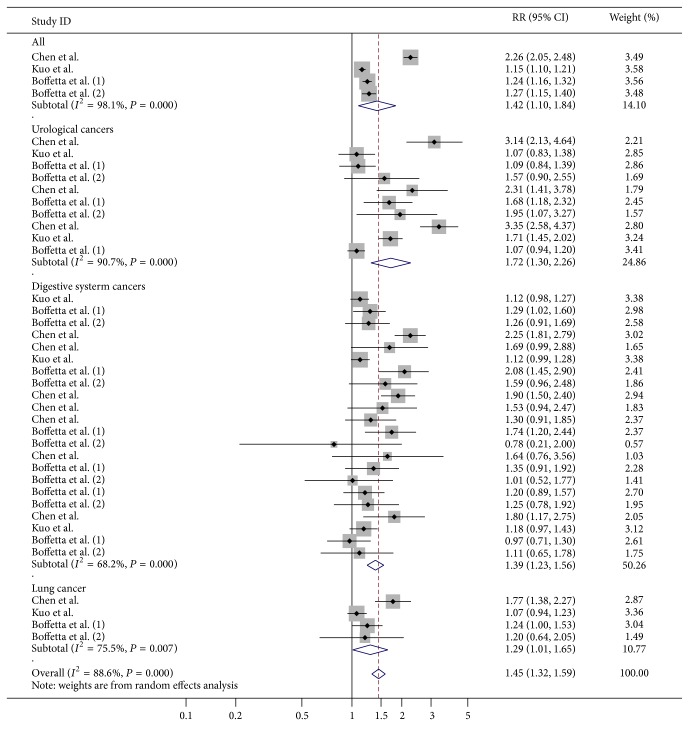
Forest plot for gout and cancer risk.

Gout was related to a significantly increased risk of cancer as suggested by overall analysis of four independent cohort studies (RR = 1.42, 95% CI 1.09–1.84, *P* = 0.008) (Table 2, Figure 1). However, the between-study heterogeneity was significant (*I* ^2^ = 98.1%, *P* < 0.001). Besides, no significant association between gout and total cancer risk was observed in the male population, suggested by the pooled RR (RR = 1.67, 95% CI 0.93–3.01, *P* = 0.087) (Table 2). Sensitivity analysis did not alter the pooled results (data not shown).

An elevated risk of urological cancers was observed to be associated with gout, although obvious heterogeneity existed among included studies (RR = 1.72, 95% CI 1.30–2.26, *P* < 0.001; *I* ^2^ = 90.7%, *P* < 0.001) (Table 2).

For the risk of digestive system cancers, the pooled RRs suggested that gout exerted risk effect on the development of digestive system cancers (RR = 1.39, 95% CI 1.23–1.56, *P* < 0.001; *I* ^2^ = 68.2%, *P* < 0.001) (Table 2).

A modest association between gout and lung cancer risk was demonstrated, which suggested a risk factor of gout for the development of lung cancer (RR = 1.29, 95% CI 1.01–1.65, *P* = 0.039; *I* ^2^ = 75.5%, *P* = 0.007) (Table 2).

No significant relationship between gout and the risk of breast cancer or brain cancer was observed in our study (Table 2).

### 3.3. Publication Bias

Begg's funnel plots and Egger's test did not show any evidence for publication bias risk in the present meta-analysis (data not shown).

## 4. Discussion

The present meta-analysis based on three prospective cohort studies shows the evidence that gout confers risk effect on carcinogenesis, particularly in urological cancers, digestive system cancers, and lung cancer. Besides, gout did not modify the risk of breast cancer or brain cancer or total cancer risk in males, as suggested by the pooled RRs, respectively. Nevertheless, the findings about breast and brain cancers risk must be interpreted with caution due to limited sample size. Sensitivity analysis did not materially alter the pooled results.

Gout is a disorder of purine metabolism, which has been suggested in relation to many kinds of diseases, such as end-stage renal disease, myocardial infarction, obesity, and diabetes, due to increased serum uric acid [21–24]. Kuo et al. have reported that gout is an independent risk factor for myocardial infarction even in young people and those without cardiovascular risk factors [22]. Gout patients aged 50 years or above were more likely to die from cardiovascular diseases than those without gout, although they had no preceding serious cardiovascular diseases [25]. Therefore, gout is positively associated with cardiovascular disease risk and mortality. Hyperuricemia is a common clinical presentation of gout and is usually attributed to high cell turnover and accelerated purine breakdown. Despite the antioxidant properties of uric acid in preventing the formation of carcinogenic oxygen radicals, little evidence supports its role in protecting against cancer [8, 12, 26]. Hyperuricemia is a rare complication in cancer and is especially common in patients with hematological malignancies, for instance, acute lymphocytic leukemia and Burkitt lymphoma [27, 28]. Given the hypothesis of link between metabolism syndrome and cancer, many studies have investigated roles of serum uric acid, hyperuricemia, and gout in carcinogenesis and cancer mortality [6, 7, 13, 20, 29, 30]. Kolonel and colleagues firstly elucidated that high level of serum uric acid exerted risk effect on prostate carcinogenesis, but not the development of overall cancer and cancers of stomach, colon, rectum, lung, bladder, or hematopoietic system [31]. Serum uric acid was not an independent risk factor for cancer mortality; however, it could predispose diabetes to die, which suggested an interactive effect of uric acid with diabetes on the risk of death from cancer [32]. Similarly, hyperuricemia was not associated with the RRs of cancer-related death [30]. Taken together, the effect of serum uric acid on cancer risk and mortality remains inconclusive.

Up till now, three prospective cohort studies with a total of 50,358 subjects have been performed to evaluate the association between gout and cancer risk [6, 13, 20]. Nonetheless, the findings were conflicting and inconclusive. Boffetta et al. reported that gout patients were at an elevated risk of overall cancer and cancers of the oral cavity and pharynx, colon, liver and biliary tract, pancreas, lung, skin, endometrium, and kidney, as well as of malignant melanoma among both males and females [20]. The study provided no evidence of a protective role of serum uric acid in carcinogenesis [20]. The findings demonstrated by Kuo et al. were consistent with those of Boffetta et al. [6]. However, no increased risk of liver, colon, lung, stomach, bladder, and breast cancers was observed in gout patients, respectively [6]. The study by Chen et al. showed that gout patients were more likely to develop most cancers including cancers of prostate, bladder, kidney, colorectum, liver, gallbladder and biliary tract, lung, and stomach [13]. The significant association between gout and increased risk of overall cancer in those studies was not likely generated due to chance. Nevertheless, concerning the risk of individual cancers, bias might be introduced because of small sample size of certain cases. We performed a meta-analysis of all published studies in relation to the role of gout in cancer risk. The pooled RRs suggested that gout was a risk factor for carcinogenesis, especially the development of urological cancers, digestive system cancers, and lung cancer. The effect of gout on brain cancer and breast cancer warrants further investigation by more epidemiological studies with large sample size.

## 5. Limitations

Some limitations must be seriously considered. Firstly, only three cohort studies were included into our study, but the statistical power was sufficient in estimating the role of gout in carcinogenesis. More relevant studies on the association between gout and individual cancers risk, especially brain and breast cancers, are warranted for further investigation. Secondly, adjusted factors of all included studies were not consistent. Thus, bias might be introduced in our study because of confounding factors such as age, sex, and ethnicity. Future studies based on adjusted analysis are encouraged to investigate the effect of gout disease on cancer risk. Thirdly, the influence of gout disease duration in tumorigenesis was elucidated in a recent study [13]. We failed to estimate cancer risk related to the duration of gout, because relevant epidemiological studies were limited. Last but not the least, cancer is a multifactorial disease with complex pathogenic factors including genetic polymorphisms, environmental carcinogens, and history of family as well as chronic inflammatory diseases [33]. An interactive effect between gout and other carcinogenic factors may be critical in carcinogenesis, which warrants further elucidation.

## 6. Conclusions

In summary, our study does show evidence for the hypothesis of a link between gout and carcinogenesis. It suggests that gout is an independent risk factor for the incidence of total cancer, particularly urological cancers, digestive system cancers, and lung cancer. The precise association between gout and individual cancers warrants further investigation by more epidemiological studies with high quality.

## Figures and Tables

**Table 1:** Characteristics of all studies.

Study	Year	Origins	Number of subjects	Follow-up duration (years)	Baseline time	Mean age of patients	Cancer sites	Adjusted factors
Chen et al. [13]	2014	Taiwan	9,413	8	1998–2000	51.03 ± 14.52	Prostate, bladder, kidney, colorectum, liver, gallbladder and bile duct, stomach, lung, esophageal, pancreas, nasopharyngeal, brain, and oral, and so forth	Age, sex, year, and month of first diagnosis
Kuo et al. [6]	2012	Taiwan	24,088	7.8-7.9	2000–2008	42.3 ± 16.3	Prostate, bladder, colon, liver, stomach, lung, and breast, and so forth	Age and sex
Bofetta et al. [20] Men	2009	Sweden	10,500	NR	1965–1995	NR	Prostate, bladder, kidney, rectum, colon, liver and bile duct, stomach, lung, pancreas, brain, and oral, and so forth	Sex, time since first hospitalization, and gout as primary or the only diagnosis
Bofetta et al. [20] Women	2009	Sweden	6,357	NR	1965–1995	NR	Bladder, kidney, rectum, colon, liver and bile duct, stomach, lung, pancreas, brain, oral, breast, and so forth	Sex, time since first hospitalization, and gout as primary or the only diagnosis

**Table 2:** Summary of meta-analysis results.

Type of cancer	^a^RR [95% CI]	*P* value for pooled analysis	*I* ^2^ (%)	*P* value for heterogeneity analysis
All	1.42 [1.09–1.84]	0.008	98.1	<0.001
Males	1.67 [0.93–3.01]	0.087	99.0	<0.001
Urological cancers	1.72 [1.30–2.26]	<0.001	90.7	<0.001
Digestive system cancers	1.39 [1.23–1.56]	<0.001	68.2	<0.001
Lung cancer	1.29 [1.01–1.65]	0.039	75.5	0.007
Breast cancer	0.92 [0.77–1.09]	0.336	45.9	0.174
Brain cancer	1.24 [0.82–1.87]	0.309	0.0	0.946

^a^RR, relative risk; 95% CI, 95% confidence interval.

## Abbreviations

RRs: Relative risks
ORs: Odds ratio
HRs: Hazard ratios
95% CIs: 95% confidence intervals.
